# Consequences of Diabetes Mellitus in Bone Health: Traditional Review

**DOI:** 10.7759/cureus.13820

**Published:** 2021-03-11

**Authors:** Cheena Kumari, Ghozlan Yagoub, Mariam Ashfaque, Sobia Jawed, Pousette Hamid

**Affiliations:** 1 Internal Medicine, California Institute of Behavioral Neurosciences and Psychology, Fairfield, USA; 2 Neurology, California Institute of Behavioral Neurosciences and Psychology, Fairfield, USA

**Keywords:** diabetes mellitus, osteoporosis, fracture

## Abstract

The diabetes mellitus (DM) pandemic was mostly related to the growing incidence of osteoporosis worldwide. Thus, DM-induced bone fragility was recently reported as a diabetic complication. This disorder needs to be identified and diagnosed early and adequately to avoid more symptoms and impairments. Bone weight is lowered and the risk of fractures rises in type 1 diabetes mellitus (T1DM). However, type 2 diabetes mellitus (T2DM) will increase bone density per se because of the elevated chance of fracturing. This indicates that bone consistency plays an important part in the pathogenesis of diseases. This research is aimed at defining the function of advanced glycation end-products (AGEs), micro-architectural changes, and altered bone turnover. The risk of fracture can be varied by drugs used for treating DM. Thiazolidinedione exacerbates bone degradation, for example, which raises the risk of fractures, particularly in older females. In contrast, metformin and sulfonylureas appeared to have no adverse effects on bone health and could guard against fragility. Evaluating bone mineral density (BMD) and other risk factors may aid in developing tailor-made recovery plans as part of the diagnostic process. Increased osteoporosis awareness is important, considering the increasing and older population of DM patients.

## Introduction and background

Diabetes and osteoporosis are common diseases and can occur at the same time. Studies have shown that osteoporosis and diabetic fractures are more likely than chance can expect [1]. Osteoporosis is a bone disorder characterized by a decline in the overall consistency of the bone and may eventually lead to an increased risk of fractures. With age, the prevalence of osteoporosis continues to increase. Osteoporosis-related fractures have affected people above the age of 50, one-third of the female population, and one-fifth of the male population [2]. Diabetes mellitus, more commonly called diabetes, is a debilitating, long-term (or 'chronic') disease that occurs when glucose levels increase in a person's blood because their body is unable to produce any or enough of the hormone insulin, or are unable to use the insulin it produces effectively [3]. There are currently an estimated 463 million diabetic adults aged 20-79 years. This age group comprises 9.3% of the world population. The total figure is expected to rise up to 578 million (10.2%) by 2030 and by 2045 to 700 million (10.9%) [3].

Diabetes influences the functioning of many organs in the human body, including the heart, brain, kidneys, peripheral nerves, eyes, and feet. Researchers have generally accepted findings regarding the association between diabetes and osteoporosis over recent years. Both diabetes and osteoporosis are metabolic disorders with a complicated relationship [4]. Additionally, there can also be more than 100 kinds of complications involved in the disease, and it is currently known as the disease with the most complications [5]. In spite of its effects on fractures, diabetes is one of the most severe comorbidities, as suggested by studies in the USA and Europe. The presence of diabetes is individually related to an increased risk of fracture, owing to bone growth and strength improvements; the risk of hip fracture is nearly twice as high for diabetes patients as for people without diabetes [6]. In this article, we describe the mechanism of DM-induced bone fragility and fracture due to anti-diabetic medication.

## Review

Fracture prevalence in DM

Accumulated evidence has demonstrated that the risk of osteoporotic fractures with both T1DM and T2DM is considerably greater. A previous meta-analysis showed that in DM elevated probabilities of any fracture (relative risk [RR] 1.32), hip (RR 1.77), upper arm (RR 1.5), and ankle fractures (RR 1.24), with no effect on distal forearm (RR 1.02) and vertebral fractures (RR 1.56). Besides, the frequency of fractures in patients with T1DM was greater than T2DM at** **overall 1.24 fold, hip 3.43 fold, and ankle fracture 1.71 fold. However, no other variations among subgroups established the relationship of DM with the upper arm, ankle, vertebrae, and complete fractures differed by sex, nature of the study, and region [7]. An additional meta-analysis found a risk of hip fracture up to 6.3 times and 1.7 times higher than the non-diabetic trials of T1DM and T2DM patients, respectively. In patients with diabetes, there has been a 2.03-fold more severe risk of vertebral fracture than in non-diabetes controls. Based on a review in the integrated analysis of three large prospective future trials, the femoral neck bone mineral density (BMD) with the possibility of hip fracture was 0.59 and 0.38 higher than in non-diabetic controls for women and men with diabetes, respectively. There have been repeated recordings of Japanese men and women with T2DM an alternate vertebral fracture risk factor after age transition, lumbar BMD, and body mass index (odds ratio, men 4.7, and women 1.9) [8].

Bone mineral density in diabetes

In T2DM, bone minerals are higher than controls assessed by dual-energy X-ray absorptiometry (DXA) at the femoral neck, shoulders, and spine. In T2DM, younger, male, and higher BMI levels are correlated with a higher BMD score. Considering that in patients with T2DM fracture risk is increased due to low BMD, a decrease in bone strength or other diabetes risks may increase the risk of fractures [9].

Pathogenesis of impaired bone quality

In diabetic patients, the pathogenesis of bone quality change is most likely multifactorial: 1) deposition of advanced glycation end-products (AGEs) in the bone matrix, 2) micro-architectural changes and bone strength, and 3) serum bone turnover markers are considered necessary.

Advanced Glycation End-Products (AGES)

The bone matrix includes abundant collagens of type 1, and by forming physiological crosslinks between collagen fibers, bones can retain their flexibility and strength. AGEs are formed by the parallel, nonenzymatic chemical glycoxidation of amino protein groupings. When patients have diabetes, AGEs are formed non-physiologically. However, several studies have demonstrated a considerably higher serum AGE rate in diabetic patients than in non-diabetic [10]. With age, AGEs are known to build up in various tissues, including atherosclerotic plaques in the coronary artery, kidney, brain, and bone. A well-characterized AGE product, pentosidine is a good indicator of microvascular and macrovascular problems in diabetic patients [11]. The quantity of bone pentosidine is linked to the strength of the human spine, irrespective of BMD [12]. Increased serum pentosidine, AGEs, and soluble receptors of AGE (sRAGE), compared with the control group with T2DM, have been recorded [12]. In a recent clinical trial of T1DM patients, bone biopsy samples were taken which showed the pentosidine content of the trabecular part of the bone was substantially and positively linked to HbA1c and improved in T1DM and fracture patients. Serum and urine levels of pentosidine can be used as markers for bone strength, as circulating levels of pentosidine are correlated with cortical bone pentosidine. In adults with T2DM, serum pentosidine was associated with an increased risk of vertebral fracture, while urinary pentosidine is correlated with an increased risk of clinical and vertebral fractures [8]. New research has found that a higher level of urinary pentosidine is significantly associated with an increased incidence of clinical fracture in older patients with T2DM[8]. We also performed a cross-sectional study showing that the serum pentosidine levels in postmenopausal women with T2DM were strongly and positively associated with a prevalent vertebral fracture** **[8] Hence, the deposition of pentosidine collagen crosslinks in the bone can be a major cause of decreased BMD in patients with DM [8].

Microarchitectural Changes and Bone Strength

Because decreased changes in bone quality are studied using different techniques, using magnetic resonance imaging (MRI) assessed more significant gaps in T2DM trabecular network compared with baseline controls [12].** **Few histomorphometric details of T2DM patients are available. The peripheral high-resolution quantitative computed tomography (HRpQCT)-analyzed bone microarchitecture provides contrasting findings [13]**.** HRpQCT is a three-dimensional imaging procedure that independently measures volumetric bone mass, microarchitecture, and structure for both the cortical and trabecular compartments. Thus, HRpQCT can provide more insight into the processes that underlie bone fragility. Based on a clinical review using HRpQCT, cortical bone porosity is considered a significant factor influencing bone strength, irrespective of BMD [8]. In individual experiments, it is identical to controls (for distal radius and tibia), although, in other tests, there is a decreased cortical porosity. Analysis of bone micro indentation (measuring subperiosteal bone resistance to proximal tibia penetration as an indicator of bone strength) shows reduced bone resistance in T2DM compared to controls [13]. Because of difficulties in obtaining bone histomorphometry in clinical settings, techniques for analyzing bone structure need to be simplified and readily available. This is even more important in patients with T2DM, where BMD provides a poor marker for fracture risk.

The use of trabecular bone score (TBS) [12], a non-invasive bone microarchitecture analysis instrument based on the lumbar spine's DXA image, can indirectly evaluate the consistency of the bone. This is an indicator of measuring the cancellous distribution of the bone microstructure, used to estimate the probability of fracture independent of BMD. In this way, this estimate is expected to be implemented in the therapeutic environment soon. An earlier study evaluating 57 and 43 people with and without T2DM, respectively, showed that the TBS in patients with T2DM was significantly smaller than in those without diabetes, although the BMD was higher. For women with strong glycemic regulation (HbA1c 7.5 percent), TBS was considerably higher within the T2DM group than for those with poor control (HbA1c > 7.5 percent) [12]. This means TBS in T2DM is lower than in controls, especially in patients with an altered bone microstructure and a higher fracture risk. Considering that TBS is easily accessible, it can be a useful indicator of the risk of fracture in these cases. Currently, for fracture risk estimation, there is no validating research or simple cutoffs available, but these are very urgently required. Therefore further work appears to be required in both young T1DM patients and adults with T1DM or T2DM.

Altered Bone Turnover

Bone turnover is a twofold relationship between the process of osteoblast bone formation (creation of new bone) and the process of osteoclast bone resorption (removal of old bone) [14]. Bone markers are classified into markers of bone formation and bone resorption. Bone formation markers consist of osteocalcin (OC), bone-specific alkaline phosphatase (BSAP), alkaline phosphatase (ALP), osteoprotegerin (OPG), procollagen type 1 amino-terminal propeptide (P1NP), and procollagen type-I carboxyl-terminal propeptide (P1CP) [14]. Resorptive markers consist of N-terminal cross-linked telopeptide of type-I collagen (NTX), C-terminal cross-linked telopeptide of type-I collagen (CTX), tartrate-resistant acid phosphatase 5b (TRAcP5b), receptor activator of nuclear factor kappa beta ligand (RANKL), pyridinoline (PYR), deoxypyridinoline (DPD), hydroxyproline (HP), and sclerostin (Scl).

Many biochemical studies show that markers of bone formation, PINP and OC, and markers of bone resorption CTX and TRAcP5b, are usually diminished in T2DM, whereas BSAP and NTX are frequently normal or mildly elevated [12]. Several clinical trials and meta-analyses have shown that bone growth markers, especially serum OC, are significantly reduced in individuals with T1DM and T2DM compared to those without diabetes [8]. Moreover, serum OC levels have been reported to rise in T2DM following intensive glycemic control, although BSAP levels have been decreased. Furthermore, in patients with T2DM, the OC/BSAP ratio was strongly correlated with prevalent vertebral fractures. OC is expressed in mature osteoblasts, and BSAP is expressed in the early stage of distinct osteoblasts; thus, the risk for fracturing in patients with diabetes causing osteoblast maturation derangements. While multiple reports have indicated that bone resorption marker levels such as CTX, TRAcP5b, DPD, and NTX are similar or higher in diabetic patients than in controls [8], a recent meta-analysis has shown a marked decline in bone resorption marker rates such as CTX and TRAcP5b relative to patients without diabetes [14].

A transition between osteoblast bone formation and osteoclastic bone resorption continuously renews the bone tissue. Altered bone tissue, including AGE collagen cross-linking and microcracks, can also not be repaired when the bone remodelling process gets disrupted, contributing to bone strength deterioration. Osteocytes account for 90-95 percent of bone cells, and recent findings have shown that osteocytes perform multifunctional roles in bone remodelling orchestration by controlling the functions of osteoblast and osteoclast [15]. Sclerostin is predominantly developed by osteocytes and inhibits osteoblast differentiation and bone-forming by antagonising the canonical Wnt signalling pathway by binding 5/6 receptor-related protein to receptor receptors with low-density lipoprotein [16]. Elevated serum sclerostin concentrations are correlated with prevalent vertebral fractures in patients with T2DM irrespective of BMD and bone turnover factors [8]. Such results indicate that osteocyte dysfunction in patients with diabetes can lead to bone fragility.

Other Key factor determinants for fractures in diabetic patients: insulin status and glucose levels affect bone vasculature in diabetes and muscle contribution to skeletal impairment in diabetes [1, 17]. An increased risk of injuries, which may be associated with hypoglycaemia, or a higher number of falls due to complications of reduced eye vision, brain ischemia, and low balance due to neuropathy, could be causes for further fractures despite higher densities in T2DM. However, one research has shown that these causes do not significantly explain the increased risk of fractures other than kidney problems. The impaired function of the kidneys can have multiple adverse effects, including decreased development of activated vitamin D, secondary uremic osteodystrophy, and hyperparathyroidism. Eye impairment and neuropathy can also be linked to decreased mobility, immobilization, and thus to osteoporosis 'disuse'. Microvascular complications, as macrovascular disease, can be related to decreased blood flow to the bones. In this analysis, this is not discussed further. Hypoglycaemia per se is always intermittent and has not been associate with impaired bone strength, although it may be linked to trauma [1, 18]. 

Impact of diabetes mellitus treatment on bone metabolism and fracture risk

Metformin

Metformin is administered on a daily basis and lowers the hepatic glucose levels by inhibiting mitochondrial respiratory chain 1 while enhancing insulin sensitivity and facilitating glucose absorption through the activation of active protein kinase (AMP) [19]. A randomized, longitudinal, double-blind, multicenter study compared the effectiveness and safety of long-term (80-week) glycemic control and BMD treatment of rosiglitazone/metformin with metformin monotherapy in drug-naive patients with T2DM. Following the survey, the metformin-treated population experienced slight improvements in BMD [20]. The effect of different dosages of metformin on BMD and bone metabolism in older, male patients with T2DM has been explored in new studies. The results showed that the therapeutic efficacy between the two classes did not differ greatly (P > 0.05). There was no substantial change in BMD and bone metabolism marker concentrations between the two classes (P > 0.05) before treatment. However, BMD and concentrations of bone metabolism markers improved following therapy in these two groups. In particular, in the experimental population, the levels of BMD and 25-hydroxyvitamin D were higher than in the control group, and in the experimental group, the levels of N-terminal midfragment and β-isomerized C-terminal telopeptide were lower than in the control group (all P < 0.05). High doses of metformin have been shown to help improve osteoporosis and blood glucose control in older male patients with T2DM [21].

However, in younger adults who have minimized the occurrence of bone fractures and diminished co-morbidities, metformin is commonly used. Such observations indicate another possible pathway for strengthening and preserving the integrity of the bone. These results, in summary, indicate that metformin will support the bone.

Sulfonylureas

Sulfonylureas, which owe their name to their form of phenyl-sulfonylurea, induce enhanced insulin secretion by binding to the adenosine triphosphate (ATP)-dependent K+ channel on the pancreatic beta-cell membrane. Sulfonylureas have been shown to be rational substitutes, as commonly used hypoglycemic antagonists. Data was collected to demonstrate that T2DM is associated with a higher risk of fracture fragility, independent of BMD [22]. A meta-analysis of 11 studies evaluating the role of sulfonylurea on the risk of fracture in more than 255,644 patients with T2DM showed that current sulfonylurea use was associated with a 14% increase in the risk of fracture [23]. In terms of bone metabolism, the function of sulfonylurea is still unsure. One of those side effects was hypoglycemia in patients undergoing sulfonylurea [23]. We thus infer that hypoglycemia and an increased risk of falling may be the cause of the sulfonylurea-induced fractures. The immediate impact of sulfonylurea at bone level was removed in preclinical studies and a healthy alternative was thus presumed. More recent evidence, however, shows that the risk of hip fracture in treated patients is almost double, likely because of higher hypoglycemic rates [24].

Thiazolidinedione

Thiazolidinedione (TZD), which includes rosiglitazone and pioglitazone, facilitates the absorption of insulin and is commonly used for T2DM treatment. TZDs have been seen as important in other types of insulin resistance, such as polycystic ovarian syndrome, in addition to the documented use of T2DM therapy. Activation of the peroxisome proliferator-activated receptor (PPAR) nuclear hormone receptor, particularly in adipocytes where PPAR expression is high, mediates the effects of TZDs. In terms of increasing insulin strength in adipose tissue, mature adipocytes and triglyceride aggregation by fat droplets help pre-adipocyte differentiation [25]. The current use of TZDs was associated with an increased risk of hip fracture in a cohort study involving more than 5,000 patients with T2D [26]. In a randomized, double-blind, placebo-controlled trial, treatment with pioglitazone slightly improved the risk of fracture compared to placebo [20]. In a population-based study and meta-analysis, the risk increase has been reported, although the effect on bone appears to be more serious in women than in men [26]. A review of ADOPT (A Diabetic Outcome Progression Trial) found the adverse effects while the number of men with fractures in the rosiglitazone class did not differ from that of the other treatment groups, there were more women with fractures in the upper limb and foot in the rosiglitazone category. Further analysis and review found that fracture frequency per 100 person-years was 2.74 for rosiglitazone, 1.54 for metformin, and 1.29 for glyburide. The investigators found that long-term rosiglitazone treatment in both premenopausal and postmenopausal women, but not in men, was associated with an increased risk of fracture [27].

Incretin-Based Therapies

Two gastrointestinal hormones - Intestine Secretion Insulin - which are secreted in response to food consumption, are glucose-dependent insulinotropic polypeptide (GIP) and glucagon-like peptide-1 (GLP-1). Two different therapeutic options have been identified to compensate for the low incretin effect in T2DM patients, either by inhibiting dipeptidyl dipeptidase-4 (DPP-4), an enzyme that rapidly inactivates GIP and GLP-1 (sitagliptin, vildagliptin, saxagliptin, linagliptin, alogliptin) or by inhibiting the mimetic drug GLP-1 (liraglutide, exenatide, dulaglutide, albiglutide, lixisenatide). These drugs improve the regulation of glucose with a low risk of hypoglycemic reactions and can be used efficiently over for long term [25]. Several clinical trials have shown us that these incretins can have a beneficial impact on bone mass and strength, and recent studies have revealed that GLP-1 receptor agonists (GLP1RA) and DPP-4 inhibitors do not have a substantial clinical impact on skeletal tissue. In comparison, new epidemiological studies have shown that the use of DPP-4 inhibitors in tandem with metformin in patients with T2DM has been associated with a decreased risk of fractures [8]. The serum markers of calcium homeostasis (ALP, calcium, and phosphate) remained unchanged during exenatide therapy in one clinical trial. In addition, no substantial connection between the use of GLP1RAs and the risk of fracture in T2DM in humans was observed in a recent meta-analysis. Case-control and meta-analytical analyses and randomized controlled trials, involving patients treated with GLP-1, found little effect on the likelihood of fracture [26]. However, there is evidence that various GLP-1s may have counter effects on the likelihood of fracture in patients treated with exenatide or liraglutide, which appear to increase or decrease, respectively [28].

Sodium-Glucose Co-transporter 2 Inhibitors

These latest generations of pharmaceutical drugs selectively inhibit renal sodium-glucose cotransporter 2 (SGLT2), increasing urinary glucose excretion, and hence the initial three on the market SGLT2 inhibitors, canagliflozin, empagliflozin, and dapagliflozin, were found to be of substantial reduction of bone density as well as anti-diabetic activity [29, 30]. In subjects treated with dapagliflozin, serum phosphate, magnesium, and parathyroid hormone (PTH) were elevated relative to placebo, but no analysis indicated a baseline rise in bone turnover markers and BMD after 50 weeks of treatment with dapagliflozin, including a small improvement in serum phosphate and magnesium compared to placebo [31]. On the other hand, in a sample of 252 T2DM participants with mild renal dysfunction treated with dapagliflozin or placebo for 104 weeks, low-trauma fractures were observed in 6% and 9.4% of patients with 5 and 10 mg dapagliflozin respectively, while no bone fractures were found in the placebo group [31]. In addition, after 52 weeks of canagliflozin treatment in a population of >700 older patients, a new Food and Drug Administration (FDA) health review has found that BMD in the hip and lumbar spine has decreased significantly [32].

Insulin

Due to insulin resistance, T2DM is characterized by elevated serum glucose and insulin levels. With disease progression, the supply of insulin is declining and patients must be treated with insulin additionally. Standard insulin therapy for T2DM rats enhanced glycemic control, but did not boost trabecular bone density, and cortical bone mass increased. Furthermore, bone defect regeneration has risen to a control level after administration of insulin [33]. Insulin is associated with a 1.4 to 2-fold increase in fracture risk relative to no insulin use and a 1.6-fold increase in risk due to metformin monotherapy. Not all studies, however, point to a negative effect of insulin on the risk of fracture. Considering the anabolic effect of insulin on the bone, the link between insulin and a high risk of fracture may be attributable to an elevated risk of falls and hypoglycemic episodes combined with insulin therapy [26]. The findings from the HABC (Health, Aging, and Body Composition) study indicated an elevated risk of declines in insulin-treated patients at ≤6% HbA1c levels. More vigorous therapy interventions in elderly people can increase the rate of hypoglycemic events and, in turn, the risk of falls and fractures [4]. Hypoglycemia, associated with falls and, subsequently, bone breaks, is also a concern to insulin-treated patients. Insulin raises the likelihood of declines in the elderly [34].

## Conclusions

Data shows a multifactorial chance of fractures in DM. The increased risk arises from many factors. The patient should screen for osteoporosis in concern that diabetic patients at a higher risk of fracture. Pentosidine has a significant role to play in altering bone strength. The TBS satisfies the criteria for a non-invasive bone microarchitecture measurement technique, a key bone strength determinant. Due to microvascular complications of DM, it’s important to maintain adequate glycemic control to decrease the fracture risk. Anti-diabetic drugs also affect bone metabolism. Thiazolidinediones, the most affected drug on bone, and metformin have a preventive role in bone degradation. The fracture can be indirectly occurring with sulfonylurea due to falling in hypoglycemia. Optimal glycemic regulation should be sought, but the particular (sometimes detrimental) impact of some antidiabetics on fracture incidence must also be recognized. Further clinical data are required to confirm the risk prevention impact of fractures in the available management strategies. 
